# Using healthcare failure mode and effect analysis as a method of vaginal birth after caesarean section management

**DOI:** 10.1111/jocn.15069

**Published:** 2019-10-23

**Authors:** Ying Liu, Wei Zhu, Shiguan Le, Wenxian Wu, Qun Huang, Weiwei Cheng

**Affiliations:** ^1^ International Peace Maternity and Child Health Hospital Shanghai Jiao Tong University School of Medicine Shanghai China; ^2^ Shanghai Key Laboratory of Embryo Original Diseases Shanghai China; ^3^ Institute of Embryo‐Fetal Original Adult Disease Affiliated to Shanghai Jiao Tong University School of Medicine Shanghai China; ^4^ Department of Surgery and War Surgery Shanghai Changzheng Hospital Second Military Medical University Shanghai China

**Keywords:** caesarean section, foetal monitoring, healthcare failure mode and effect analysis, obstetric labour complications, vaginal birth after caesarean

## Abstract

**Aims and objectives:**

This research was conducted to explore the effectiveness of employing the healthcare failure mode and effect analysis method in the management of trial of labour after caesarean, with the aims of increasing vaginal birth after caesarean section rate and reducing potential risks that might cause severe complications.

**Background:**

Previously high caesarean section rate in China and the “two children” policy leads to the situation where multiparas are faced with the choice of another caesarean or trial of labour after caesarean. Despite evidences showing the benefits of vaginal birth after caesarean, obstetricians and midwives in China tend to be conservative due to limited experience and insufficient clinical routines. Thus, its management needs further optimisation in order to make the practice safe and sound.

**Design:**

A prospective quality improvement programme using the healthcare failure mode and effect analysis.

**Methods:**

With the structured methodology of healthcare failure mode and effect analysis, we determined core processes of antepartum and intrapartum management, conducted risk priority numbers and devised remedial protocols for failure modes with high risks. The programme was then implemented as a clinical routine under the agreement of the institutional review board and vaginal birth after caesarean success rates were compared before and after the quality improvement programme, both descriptively and statistically. Standards for Quality Improvement Reporting Excellence 2.0 checklist was chosen on reporting the study process.

**Results:**

Seventy failure modes in seven core processes were identified in the management process, with 14 redressed for actions. The 1‐year follow‐up trial of labour after caesarean and vaginal birth after caesarean rate was increased compared with the previous 3 years, with a vaginal birth after caesarean rate of 86.36%, whereas the incidence of uterine rupture was not compromised.

**Conclusions:**

The application of healthcare failure mode and effect analysis can not only promote trial of labour after caesarean and vaginal birth after caesarean rate, but also maintaining a low risk of uterine rupture.

**Relevance to clinical practice:**

This modified vaginal birth after caesarean management protocol has been shown effective in increasing its successful rate, which can be continued for further comparison of severe complications to the previous practice.

**What does this paper contribute to the wider global clinical community?**

- Healthcare failure mode and effect analysis method as a quality improvement tool that can help procedure management in vaginal birth after caesarean (VBAC).
- The revised protocol provides sound support for obstetrician’s and midwife’s practice in taking care of VBAC population.

## 1 BACKGROUND

A research conducted by Lumbiganon et al. (2010) showed a caesarean section (C‐S) rate of 46.2% in mainland China, which is to some extent caused by the “one‐child policy.” However, the overall abandonment of “one‐child policy” in mainland China since 2015 confronted numerous women of previous C‐S with the choice between trial of labour after caesarean (TOLAC) and elective repeat caesarean section (ERCS). It was reported that in a tertiary maternity hospital in Shanghai, the rate of repeated C‐S reached over 90%, ranking the top among all C‐S indicators (Shi & Zhang, 2016). Another retrospective study (Minsart, Liu, Moffett, Chen, & Ji, 2016) conducted in Shanghai showed that only 77 out of 368 (20.9%) women with one previous C‐S had a vaginal birth. Yet in developed countries, vaginal birth after caesarean (VBAC) rate in the population of one previous C‐S without previous vaginal birth is 72%–75% (RCOG, 2015). The choice of VBAC depends much on the obstetricians’ inclination in China under the traditional concepts of Chinese people and the likelihood of the intense physician–patient relationship as well. Relatively limited experience on its management refrained obstetricians and midwives from TOLAC. Thus, to determine the best choice of delivery for mothers who have experienced C‐S and guarantee their safety become one of the most urgent missions that obstetrical staffs need to accomplish in mainland China.

## 2 LITERATURE REVIEW

Trial of labour after caesarean provides women the possibility of VBAC delivery, which is defined by vaginal delivery by a woman with a history of a previous caesarean delivery (ACOG, 2017). According to Landon et al. (2004), TOLAC is associated with a greater perinatal risk than in ERCS without labour, although absolute risks are low. The estimated incidence of uterine rupture was reported to be approximately 1 in 500 women planning VBAC and 1 in 1,000 women planning an ERCS (Spong, 2012). Whereas a systematic review (Santhi Sri & Xiang, 2016) including 17,598 successful cases found no significant difference in the incidence and relative risk of adverse maternal outcomes between the VBAC and ERCS groups, such as the rate of postpartum haemorrhage, blood transfusion and hysterectomy. Nevertheless, some guidelines have proposed the benefits and risks of VBAC and ERCS, and suggested individualised careful consideration by both healthcare providers and the mothers themselves (ACOG, 2017; QCL, 2015; RCOG, 2015).

The Royal College of Obstetricians and Gynaecologists suggested that successful VBAC had the fewest complications, for which the chance of success or failure was an important consideration when choosing the mode of delivery (RCOG, 2015). Internationally, there have been some predictive models through which the success rate of VBAC can be calculated, with the accuracy of each model varied (Flamm & Geiger, 1997; Gonen, Tamir, Degani, & Ohel, 2004; Grobman et al., 2007; Smith, White, Pell, & Dobbie, 2005). Some of the models with better sensitivity included gender as an indicator, whereas gender testing is illegal in China. Therefore, some researchers are focusing on establishing a domestic VBAC score system (Xing, Qi, Wang, & Yang, 2019), providing some guidance for clinical practice.

Although efforts have been made to promote VBAC outcomes, the management of TOLAC process, especially during the first and second stage of labour, requires careful inspection since adverse event can be caused by healthcare management rather than clients’ conditions. Clinical risk management consists of complex actions done to improve the quality of care provided by healthcare organisations and to assure clients’ safety (Bonfant et al., 2010). In fact, the provision of VBAC poses considerable challenges for obstetricians, midwives and nurse, much in the same way any high‐risk procedure may. Thus, facilities must introspect their capacity to deal with VBAC‐related complications in a safe and timely fashion. Yet due to the preliminary period of VBAC practice, its management in mainland China varies tremendously: devoid of guidelines for developing and implementing a VBAC programme induces the management process with potential threats of severe complications.

Failure mode and effective analysis (FMEA) is an effective approach to manage risks originated in 1950, based on which the application in healthcare practice was implemented. The healthcare failure mode and effect analysis (HFMEA), simplified from FMEA, has been deemed as a proactive way to identify vulnerabilities in a care system and deal with them effectively (Stalhandske, DeRosier, Wilson, & Murphy, 2009). It differs from FMEA in application area, RPN calculation and prioritisation of failure modes. Since 2003, HFMEA has been recommended as a standard of the Joint Commission on the Accreditation of Healthcare Organizations (JCAHO) for proactive risk assessment. Allowing for narrow anticipation for some emergent and critical conditions in delivery, we employed this method for process management in order to identify high risks in present VBAC management and develop coping strategies, thereby controlling the incidence of adverse outcomes that might be correlated to potential errors, as well as helping more women to fulfil their expectations of VBAC.

In this research, we are aimed at developing a practical quality improvement programme to minimise the potential risks of TOLAC, as well as living up to the expectations of some multigravida with the experience of previous C‐S.

## 3 METHODS

### 3.1 Study design

The project was a prospective quality improvement programme using the healthcare failure mode and effect analysis method, with Standards for Quality Improvement Reporting Excellence 2.0 (SQUIRE 2.0) checklist (Ogrinc et al., 2016) chosen for report (see File S1). Comparing to previous data, this study cannot be randomised.

The project was conducted between January 2016–March 2016 in a tertiary maternal and child hospital in Shanghai. In 2015 only, the total number of pregnancies with previous C‐S was 1,760, of which 51 cases were vaginal birth, with a VBAC rate of 2.9%. Specifically, only those with one previous C‐S were allowed for TOLAC, in order to minimise potential risks.

Under institutional regulations, these women were admitted to the obstetrical ward as signs of labour occurring and transferred to the delivery room (LDR) when uterine contractions came regular. The complex management process was inspected by a multidisciplinary team to identify high‐risk segments; thus, caseload care measures were developed to assure safe delivery.

### 3.2 Data collection

#### 3.2.1 HFMEA steps

##### Creation of an HFMEA group

The focus group consisted of eight members, including two senior obstetricians, two senior midwives, an anaesthetist, a nutritionist, a medical administrator and a senior nurse manager. The clinical staffs had a minimum of 10‐year working experience and intermediate title exclusively, whereas the two supervisors both at least 5‐year management experience. All members participated in a systematic HFMEA training programme for totally 30 hr and were tested to check for mastery of basic knowledge.

##### Development of process maps

After two rounds of discussion, the group analysed and evaluated the prior clinic assessment flow in women with scarred uterine and selected core processes. Potential failure modes were then identified by brainstorming and recorded on the HFMEA worksheet.

##### Conduction of hazards analysis

Before evaluation, the group agreed on scoring criteria, namely the potential occurrence of a failure (O) and the severity of its potential negative impact on the overall process (S), which were consequently rated by numerical scores from 1 to 4 based on the degree of impact and frequency according to the Veterans Affair National Center for Patient Safety (VANCPS) classification (DeRosier, Stalhandske, Bagian, & Nudell, 2002). The average scores were calculated to guarantee accuracy. Then, the simplified RPN of each potential failure mode was obtained (O × S), suggesting its relevance to the process. A threshold of 8 was regarded as high‐risk step. Meanwhile, the higher RPN attached a greater priority to make remedial measures. Decision tree analysis was then conducted to determine whether those failure modes with a RPN score over 8 should be adapted for quality improvement.

##### Identify actions and outcome measures

Risk control measures were developed according to action types and potential causes of failure modes deemed as major. Besides, a specialised midwifery VBAC clinic was integrated into the revised action plans, and its running policies were developed. Due to the limited clients in previous three years before the protocol, the sample size was comparatively larger. The 1‐year follow‐up quality improvement initiation was conducted since April 2016. VBAC rate, TOLAC rate, number of emergent C‐S and the incidence of uterine rupture or dehiscence were compared descriptively before and after programme implementation. To provide a better indication of the HFMEA management impact, Chi‐square tests were conducted on VBAC success rates before and after implementation, respectively, for 2013, 2014, 2015 and the average of the three years. The inclusion criteria were revised as in the protocol.

### 3.3 Ethical consideration

This study was a quality improvement project. Since the management process of VBAC was adapted, the ethical consideration was necessary for this study. It was approved by Institutional Ethics Committee [No. (GKLW)2016‐101].

## 4 RESULTS

### 4.1 RPN scoring and decision tree analysis

**Figure 1:**
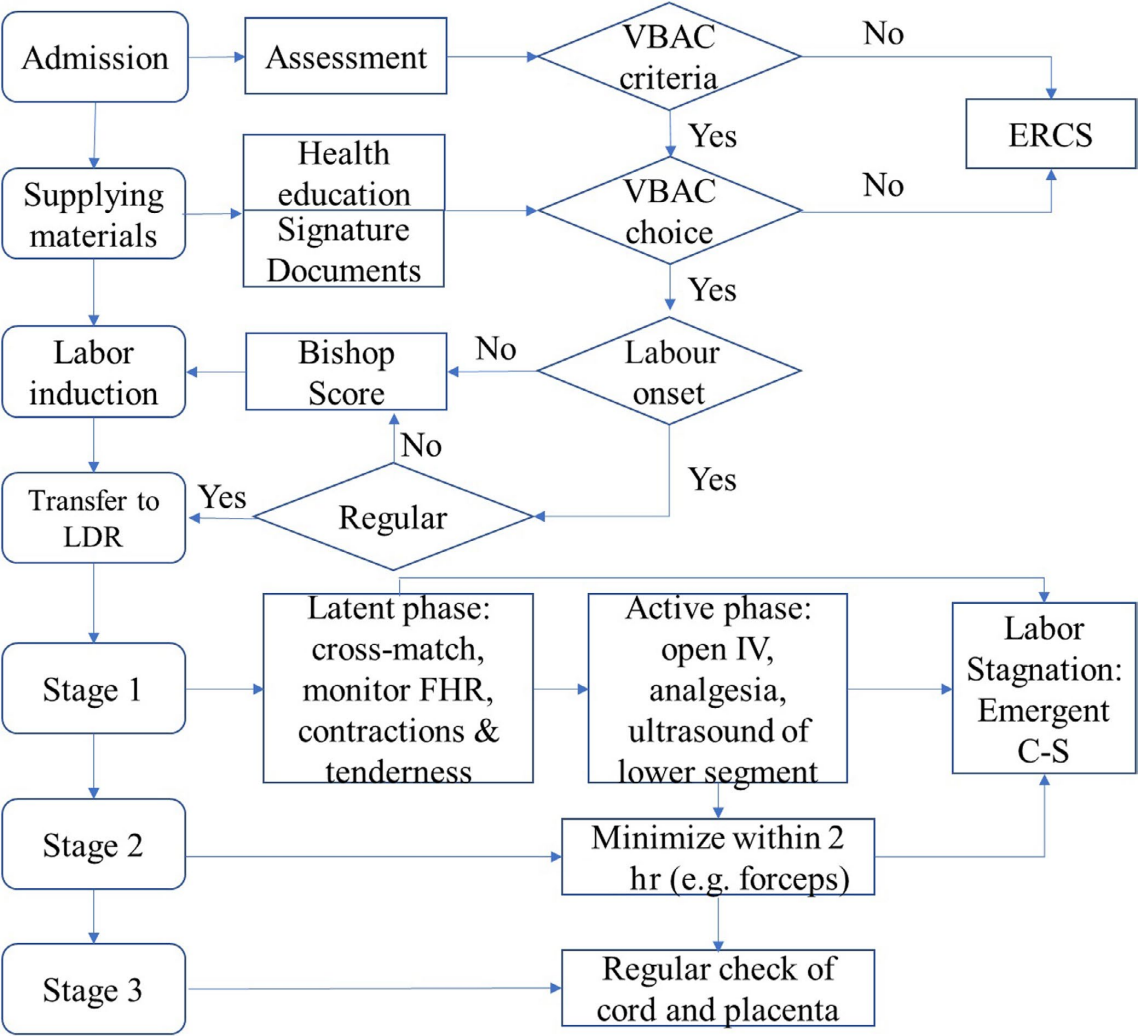
Seven core processes in vaginal birth after caesarean management [Colour figure can be viewed at http://www.wileyonlinelibrary.com]

We identified 70 failure modes in seven core processes (Figure 1), the RPN scores of which ranging widely from 1 to 12 with a total RPN of 335 (Table 1). Among these failure modes, 15 (21.4%) were considered very high risk (RPN ≥ 8), involving all seven core processes. With the decision tree analysis, 14 of the high‐risk failure modes were determined to be further proceeded. The listed failure mode (Table 2) *designated senior obstetrician in charge* had existing control measure, for which it was excluded for further action.

**Table 1:** Summary of the failure modes and RPNs in seven selected core processes

Core process	Number identified		RPN		
	Activities	Failure modes	Min	Max	Total
Admission assessment	4	9	3	12	54
Supplying materials	4	4	1	9	22
Decision of labour induction	5	7	2	12	37
Transferring to LDR	3	4	1	8	15
Stage 1 observation	12	19	1	12	93
Stage 2 management	11	16	2	12	71
Stage 3 management	9	11	2	9	43
Total	48	70			335

Abbreviation: RPN, risk priority number.

**Table 2:** Decision tree analysis of the project

Failure mode	Decision tree analysis			
	Single point weakness	Existing control measure	Detectability	Proceed
Admission assessment				
Assessment is not integrated	N	N	N	Y
VBAC indicators and contraindicators are not reviewed or updated periodically	N	N	N	Y
There is a designated senior obstetrician in charge of each individual	N	Y	Y	N
Additional screening tool to determine risk for uterine rupture	N	N	N	Y
Supplying materials				
Incomplete documentations of inform consents	N	N	N	Y
Lack of various education ways to make teaching individualised	N	N	N	Y
Decision of labour induction				
Inadequate assessment before induction	N	N	N	Y
Lack of experience of induction observation	N	N	N	Y
Transferring to LDR				
Delayed transference	N	N	N	Y
Stage 1 observation				
Fail to recognise abnormal foetal heart by midwives	N	N	N	Y
Fail to distinguish early signs of uterine rupture	N	N	N	Y
Delayed emergent C‐S when TOLAC discontinued	N	N	N	Y
Stage 2 management				
Prolonged second stage of labour	N	N	N	Y
Maternity changed will of VBAC	N	N	N	Y
Stage 3 management				
Omission of potential risk of uterine rupture or haemorrhage	N	N	N	Y

Note

N = no; Y = yes.

### 4.2 Implementation of the remedial actions

The remedial action plan was enacted for each of the 14 failure modes (Table 3). Since several failure modes were concerning staff training, a lecture and emergency drill practice was integrated into remedial actions, especially for the fresh.

**Table 3:** Remedial actions for high‐risk failure modes

Failure mode	Action type	Actions for stopping
Assessment is not integrated	C	Develop a checklist to include all necessary assessments and complete the form on client's admission
VBAC indicators and contraindicators are not reviewed or updated periodically	B	The multidisciplinary VBAC expert team annually hold a meeting to review institutional VBAC management policy VBAC client should be assessed in an individualised way
Additional screening tool to determine risk for uterine rupture	B	Include reliable VBAC risk score tool^[8]^ as additional assessment parameter
Inadequate assessment before induction	C	Develop a checklist to identify conditions where induction is not appropriate Senior obstetrician in charge are obliged to ensure assessments are completed timely
Lack of experience of induction observation	A	Institutional routine of VBAC induction is reviewed Induction is managed by middle‐level obstetricians and when contraction comes every 3–4 min, an assigned HCP should monitor the process of labour Senior midwives are obliged to assess the progress of induction periodically
Incomplete documentations of inform consents	B	Senior obstetrician in charge should check the inform consent if it is signed by the junior obstetrician Midwives are responsible for verifying VBAC inform consent and client's understanding and should hand off if unaccomplished
Lack of various education ways to make teaching individualised	B	Develop VBAC pregnancy education booklets and hand them out from the first antepartum clinic to help clients familiarise the process and potential risks Establish midwife VBAC consult clinic for clients and make birth plan together with them VBAC lecture is held monthly in the hospital as part of the *Pregnancy School Programme* Charge nurse or midwife is responsible for health education after admission and provides education materials about labour process
Delayed transference	C	a. Clients are transferred to the delivery room once labour onsets, rather than until regular contractions occur b. The head nurse in each obstetrical ward reinforce transference cautions and ensure that each client is accompanied by her nurse throughout transferring
Fail to recognise abnormal foetal heart by midwives or nurses	C	VBAC clients accept doppler auscultation with a shorter time interval routinely New nurses and midwives, as well as interns, are trained for electrical FHR monitor to guarantee basic knowledge on abnormal cases The central FHR monitor system is maintained twice a year and the hospital equipment section hotline should be available for help
Fail to distinguish early signs of uterine rupture or dehiscence	C	Train all nurses and midwives to recognise uterine rupture or dehiscence promptly and evaluate their skills periodically Reinforce client education on abnormal contraction symptoms Each client with scarred uterus will be assessed if there is any pain on the incisional sites according to institutional routine d. Intern nurses and midwives are supervised during the whole process of taking care of VBAC client in case of ignoring chief complaint
Delayed emergent C‐S when TOLAC discontinue	C	5‐min emergent C‐S drills are implemented quarterly, followed by a debriefing Case analysis is held among relevant nurses, midwives, obstetricians and managers within a week after VBAC fails
Prolonged second stage of labour	A	Active management of second stage of labour, including induction as appropriate (see induction step), assisted vaginal delivery (forceps or vacuum extraction) b. If the second stage stagnates, emergent C‐S should be implemented
Maternity change her will of VBAC	A	Follow agency rule of “calling off” through the process of VBAC The nurse, midwife and obstetrician in charge of VBAC client should be fully aware of her will and get the care team informed once the will is changed
Omission of potential risk of uterine rupture or haemorrhage	C	Routinely exploration of uterine cavity is implemented after the third stage of labour is implemented to timely discover any potential rupture or dehiscence

Abbreviations: A, elimination; B, control; C, accept; FHR, foetal heart rate; HCP, healthcare provider.

The brand‐new midwifery VBAC clinic, run by four senior midwives, was established based on extant midwifery clinic. Before the consult service, each midwife accepted a 3‐day training on VBAC knowledge in order to minimise bias. The continuous mode covers the whole process of pregnancy, which provides individualised and specified knowledge for each term. The team also drafted handout materials so that VBAC clients would have a better understanding about their choices. The VBAC clinic had a total outpatient attendance of 647 from April 2016–March 2017.

**Figure 2:**
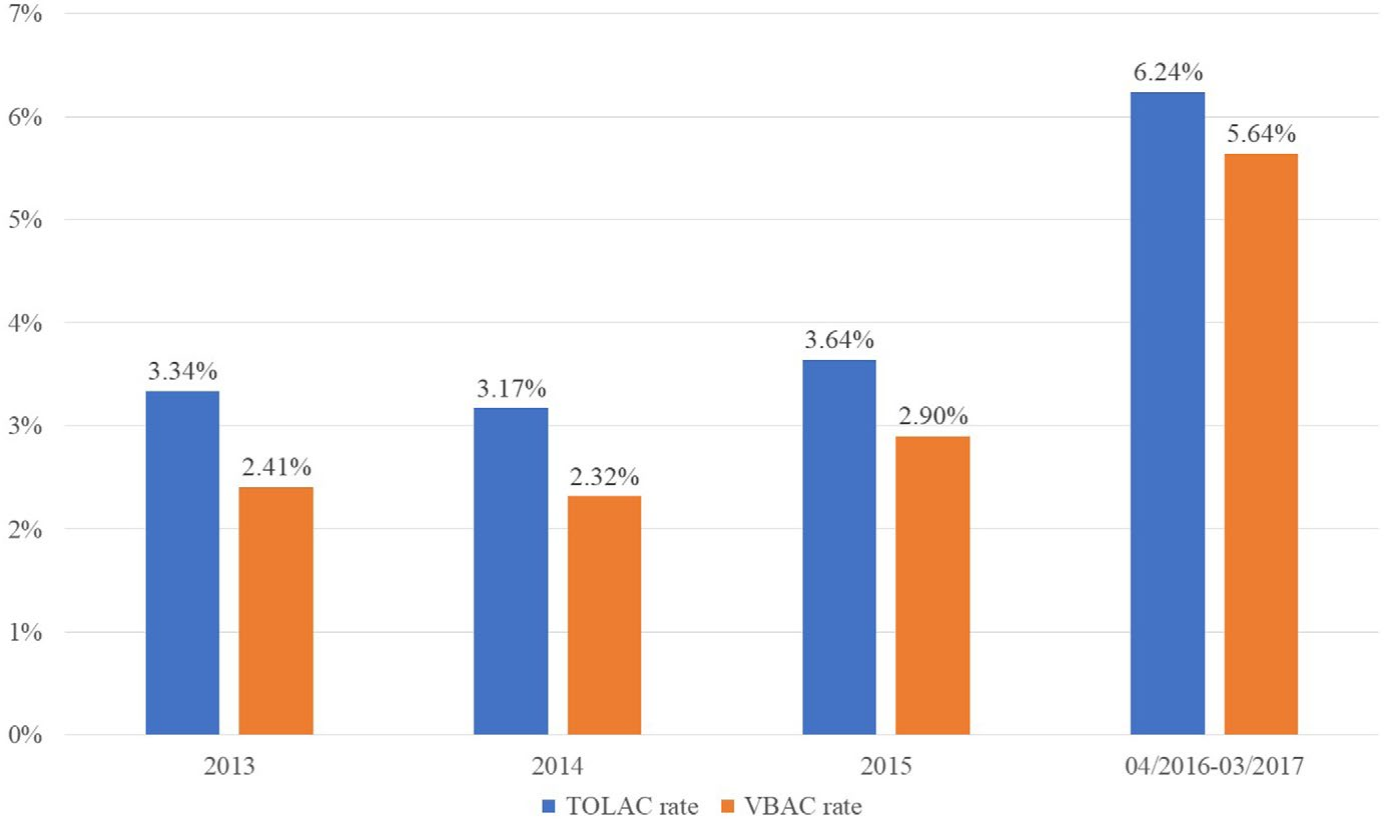
Trial of labour after caesarean and vaginal birth after caesarean rate from 2013–2015 and 1‐year follow‐up after healthcare failure mode and effect analysis [Colour figure can be viewed at http://www.wileyonlinelibrary.com]

With the introduction of the revised VBAC management process for the 1‐year follow‐up, 144 women (6.2%) among 2,306 who were pregnant with previous C‐S chose TOLAC, of which 130 cases (5.6% of all women) succeeded in VBAC, representing a success rate of 90.3% (130/144). For the 14 cases who failed, 8 tried until the active phase (i.e., cervical dilation over 3 cm). The VBAC rate increased steadily by each quarter from 3.0%–7.1%. Back to 2013, the cases of pregnancy with scarred uterine were 1,077, 1,292 and 1,760 for three successive years, and the clients accomplished VBAC were 26, 30 and 51, respectively. Both the TOLAC rate and VBAC rate ascended (Figure 2). In addition, the chi‐square tests showed significant difference between VBAC success rates before and after implementation, for the year 2014–2015, as well as the average of the three years (Table 4). In terms of the risk of uterine rupture, one case occurred in 2014, whereas uterine dehiscence occurred once in 2016 while manual exploring the uterine cavity after delivery of the placenta, with no severe maternal or neonatal complications.

**Table 4:** Chi‐square test on vaginal birth after caesarean success rates before and after implementation

	2013	2014	2015	Average of three years	04/2016−03/2017
TOLAC	36 (3.34)	41 (3.17)	64 (3.64)	47 (3.42)	110
VBAC	26 (2.41)	30 (2.32)	51 (2.90)	36 (2.62)	95
Successful TOLAC rate	72.16%	73.17%	79.69%	75.01%	86.36%
*p*‐Value	.051	.044	.000	.016	—

Note

Values are presented as numbers (percentage of all clients with once C‐S); VBAC success rates are compared before and after implementation, respectively, for 2013, 2014, 2015 and the average of the 3 years.

## 5 DISCUSSION

Not until recently has VBAC been a choice that is considered risky for many obstetrical staff and the maternity in China, the management process of which is still undergoing an exploring phase. With comparatively limited experiences, we chose the HFMEA method as a preventative way to find the underlying systems‐based problem that has been unaddressed and focused on preventing severe complications in VBAC management process. Our work, on the other hand, proved its effectiveness with yielding promising outcomes on TOLAC and VBAC rates. The steps of admission assessment, introduction of labour and distinguishing signs of uterine rupture in both stage 1 and stage 2 were deemed to have the highest RPNs, for which we devised more than one strategy to decrease the risks to the minimal.

Notably, our results show that VBAC success rate was markedly improved after the programme, with a total increase of the TOLAC rate. This indicates that more obstetricians and mothers are willing to take the option of TOLAC with a comprehensive adaptation of its management. As for the year 2013, the minor difference might be due to limited number of subjects. The larger sample size later in 2014–2015, however, provided a more reliable result. Recent domestic reports showed similar VBAC rate, ranging from 76.7%–87.2% (Li et al., 2019; Xing et al., 2019; Yang et al., 2019). In contrast to the rates reported in a literature review between 60%–77% (Tanos & Toney, 2019), the relatively higher outcome might be caused by the times of previous C‐S. For our research, only those who had once C‐S could choose TOLAC.

Conventionally, the incision way of previous C‐S, the period from last operation, devoid of comorbidities, weight and deformity of the foetal, maternal willingness, and especially the B‐type ultrasound results were taken into consideration for primary assessment. However, the introduction of VBAC risk score provided an intuitive sense on one's VBAC risk, so that the client had a better understanding on her individual situation. As the foetal gender, a predictive parameter in this tool, should be confidential to the parents under policy, we informed the parents of the risks for both male and female. Though this kind of tool has not been shown to result in improved patient outcomes (ACOG, 2017), it helped to exclude those unsuitable for VBAC with further protection. One study (Xing et al., 2019) developed a modified score system as prediction model for successful vaginal birth after caesarean delivery, showing a positive correlation with VBAC success rate with an area under the receiver operating characteristic curve of 0.849. With the occurrence of local prediction model, a comparison of the accuracy will be further conducted to help us find a better tool.

Our midwifery clinic aims at a caseload model to provide antepartum care for first, second and third trimester so that assessment and guidance can fit in the characteristics of each period. According to Whitelaw, Bhattacharya, McLernon, and Black (2014), women searching for internet on VBAC were exposed to limited information, thus providing personalised and full consult is important for the clients. Similar consult clinic was established by David, Fenwick, Bayes, and Martin (2010) to content information needs of the maternity and family, thus helping them with right decision.

Under the rudimentary stage of VBAC development in China, various attempts have been made to standardise individualised management process. On the contrary, lack of domestic evidence‐based guidelines refrained many obstetricians and midwives from the tendency of choosing TOLAC. Further studies on high‐quality clinical trials are still needed.

## 6 CONCLUSION

With the quality improvement programme using HFMEA, our study demonstrated a relatively high VBAC success rate of 86.36% in women with a previous history of C‐S. The effectiveness of applying HFMEA in TOLAC management is practical, keeping the uterine rupture outcome at low level. More evidences should be integrated into our VBAC management practice to minimise the risks.

## 7 RELEVANCE TO CLINICAL PRACTICE

This modified VBAC protocol has been shown effective in increasing its successful rate, which can be continued for further comparison of severe complications to the previous practice.

## CONFLICTS OF INTEREST

None to declare.

## AUTHORS’ CONTRIBUTIONS

(1) Study design and administrative support: Qun Huang and Weiwei Cheng; (2) Data collection: Wei Zhu and Wenxian Wu; (3) Data analysis and interpretation: Ying Liu and Shiguan Le; (4) Manuscript writing (drafting and revision): All authors; (5) Final approval of manuscript: All authors. It has been approved by all authors and has never been published.
